# Development and Progress of Balkan Medical Journal

**DOI:** 10.4274/balkanmedj.2017.5.0001

**Published:** 2017-09-29

**Authors:** Zafer Koçak, Necdet Süt, Ahmet Asan

**Affiliations:** 1 Department of Radiation Oncology, Trakya University School of Medicine, Edirne, Turkey; 2 Department of Biostatistics, Trakya University School of Medicine, Edirne, Turkey; 3 Department of Biology, Trakya University Faculty of Sciences, Edirne, Turkey

According to Journal Citation Report by Clarivate Analytics (1), the impact factor (IF) of Balkan Medical Journal arose 1.083 in 2016, which reflects 114% increase compared to the last year. This impressive progress has also changed the place from category Q4 to Q3, made Balkan Medical Journal ranked 94th among 154 general medical journals in globe and the most prestigious one in Turkey (Table 1).

This increase in IF, motivated us to make a brief analysis about the citations of the journal and its sources. We have observed that, 61% of all citations (which our journal had been referred since its integration to SCI-Expanded) appeared between January 2016 and May 2017. This analysis helped us to focus on and evaluate sources of citations.

Between January 2016 and May 3, 2017, Balkan Medical Journal had been cited 338 times by 275 journals from 32 countries (2). During this period, types of cited-articles published in the journal are shown in Figure 1. Among these; 70% are original articles, 19% are invited reviews, 7% are letters, 3% are editorial materials and remaining 1% are the others. As a summary, 89% of cited-articles consist of original articles and invited reviews.

Another topic we wonder is the IFs of journals which Balkan Medical Journal is cited. Among 275, 229 of IFs obtained from Web of Science database which was released in 2015 (2). The average IF in 2015 of these journals is 2.0±1.8 (range: 0.09-21.3). While 34.9% of the journals in which Balkan Medical Journal is cited have an IF that varies between 1-1.9, the percentage of the journals which have an IF of ≥2 is 40,7% (Figure 2).

The regional analysis of the journals in which Balkan Medical Journal is cited reveals us that our journal is predominantly cited by journals from North America (41.5%), Europe (33.5%) and Asia (12.7%) (Figure 3). Besides, it is observed that the citations stem from 51 different countries; 41.1% (113/275) are from America; 5.8% (16/275) are from United Kingdom; 5.8% (16/275) are from Turkey; 5.5% (15/275) are from Netherlands; 4.4% (12/275) are from India; and 3.6 (10/275) are from Switzerland.

In other words, while journals from North America and Europe consist 75% of total journals, the percentage of journals from Balkans which cite Balkan Medical Journal is merely 2.2%. The citation-based regional analysis (338 citations) reveals us that the leading region which cites our journal is North America (128/338, 38.2%); followed by Europe (112/338, 33.1%); and Turkey (42/338, 12.4%) (Figure 3).

We thought that it would be very interesting to check the origin of the first author of 338 papers. This analysis reveals us that the leading country is Turkey (92/338, 27.2%); followed by Asia (85/338, 25.1%), Europe (72/338, 21.3%), North America (46/338, 13.6%) and Balkan countries (20/338, 5.9%). In the country-based analysis of the first author, Chinese authors are ranked as the third after their Turkish and American colleagues with 35 studies (10.4%) (Figure 3).

In brief, 75% of the journals that cite the Journal are from North America and Europe; and similarly, 71.3% of 338 citations are from the journals published in these continents. However, the percentage of the North American and European first authors of 338 studies remain at 34.9% (Figure 3). On the other hand, Africa, Australia and New Zealand and South America are the continents where the percentage of the journals, citations, and the first authors that cite Balkan Medical Journal is below 10% (Figure 3).

One may argue that being indexed in PubMed Central in 2012 contributed and will contribute to increase the international visibility and accessibility of the journal as the last two years’ IF scores show. The rapid increase in submission allowed us to choose higher quality manuscripts. Despite recent improvements, quantity and quality of manuscripts originated from Balkan countries are still inadequate. We do believe in that, a close cooperation between team members leads the way to success. Therefore, we are in need to develop a scientific cooperation with Balkan countries. To realize this, Balkan Medical Journal aimed to constitute an efficient platform for sharing of scientific knowledge among Balkan countries.

So, factors like being cited by authors from 51 countries with higher IF journals (even IF=21) are our fuel and source of motivation. Balkan Medical Journal has now completed 38 years of publication and is well recognized by international health community. We would like to thank and express our gratitude to all members of the Editorial Board, authors, reviewers, and publisher for their contribution and tremendous support.

## Figures and Tables

**Table 1:**
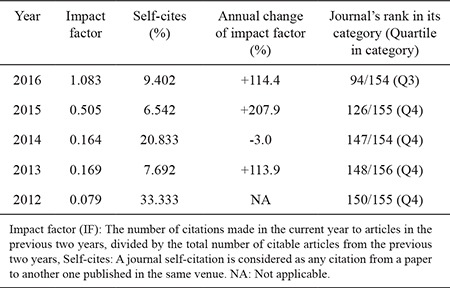
Impact factors and Balkan Medical Journal’s rank in its subject category in the last five years as published in Journal Citation Reports-JCR

**FIG. 1.**
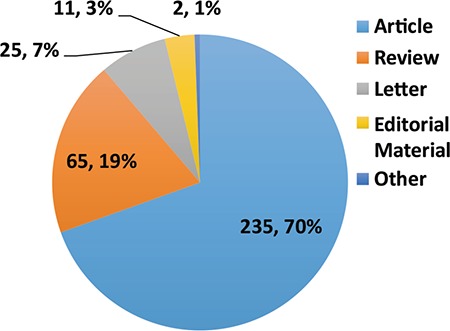
Types of cited-articles from Jan 2016 to May 2017.

**FIG. 2.**
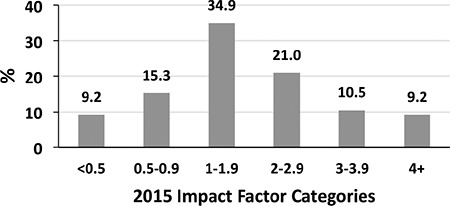
The categorical distribution of the impact factors of journals in which Balkan Medical Journal is cited from January 2016 to May 2017.

**FIG. 3.**
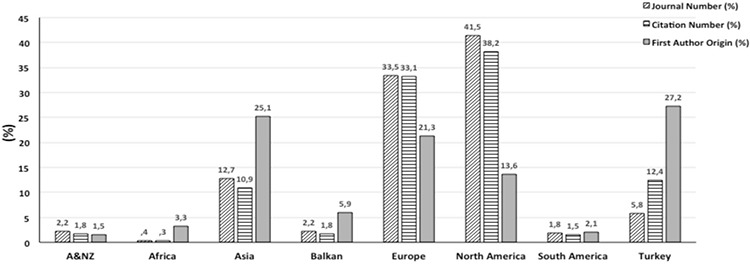
The regional distribution of the journals, citations and first author origins of articles in which Balkan Medical Journal is cited.
